# Supplementary material for: Immune Checkpoint Profiling in Humanized Breast Cancer Mice Revealed Cell-Specific LAG-3/PD-1/TIM-3 Co-Expression and Elevated PD-1/TIM-3 Secretion
Source: Cancers (Basel). 2023 May 4;15(9):2615. doi: 10.3390/cancers15092615 (PMC10177290; doi:10.3390/cancers15092615)
Supplement: Supplementary file 1 [file cancers-15-02615-s001.zip › cancers-2333141-supplementary.pdf]

Supplementary Figures

Figure S1.

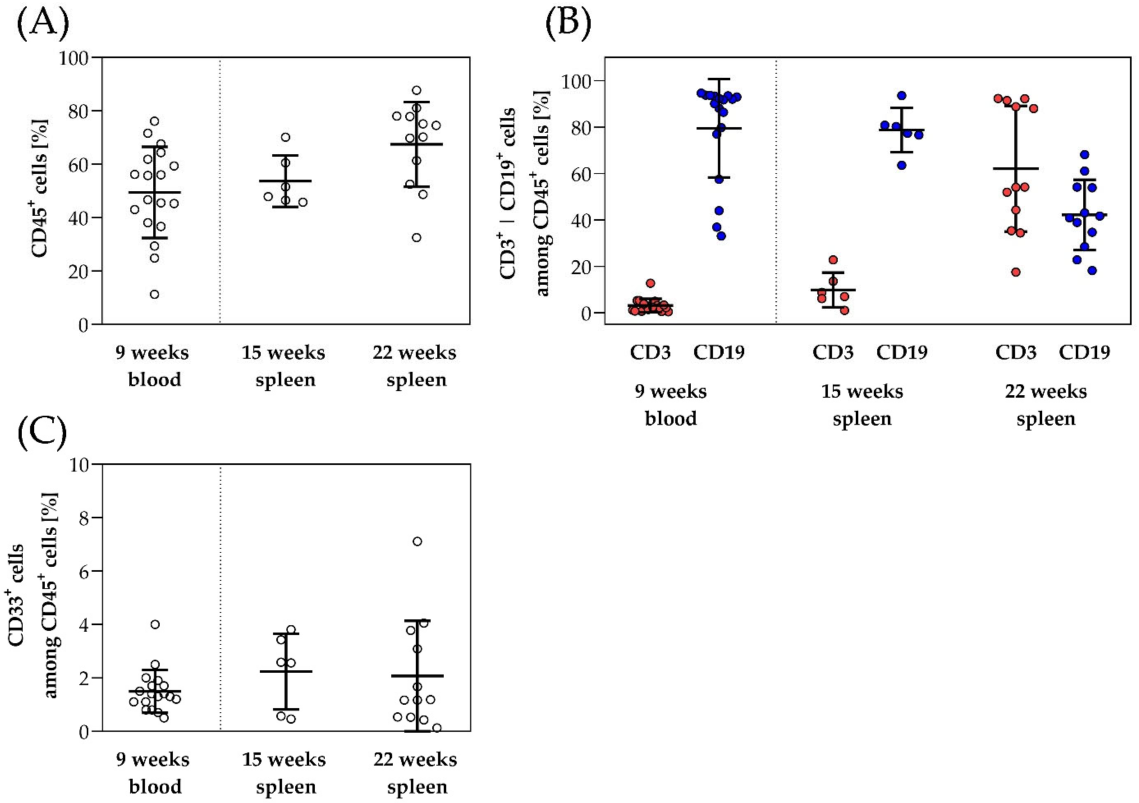

Figure S1. Engraftment success of the human immune system in mice nine weeks after transplantation and at the end of the experiment. Reconstituted leukocytes in peripheral blood in nine weeks old NSG mice were analyzed by flow cytometry JIMT-1, MDA-MB-231 and MCF-7 were transplanted orthotopically into these humanized mice one week after. At the end of the experiment, spleens of 15 weeks old (MCF-7) or 22 weeks old mice (JIMT-1, MDA-MB-231) were collected. Spleens were processed to a single cell suspension and (**A**) leukocytes ($CD45^+$), (**B**) T cells ($CD45^+$, $CD3^+$, in blue), B cells ($CD45^+$, $CD19^+$, in red) and (**C**) myeloid cells ($CD45^+$, $CD33^+$) were analyzed by flow cytometry. Each symbol represents one individual mouse (mean ± SD).

Figure S2.

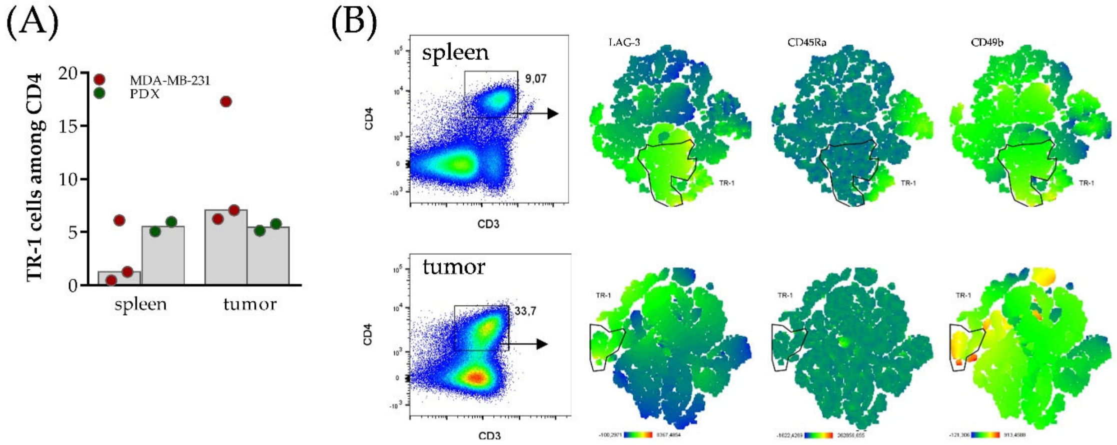

Figure S2. TR-1 CD4+ cells in HTMs. MDA-MB-231 cells or PDX tissue were transplanted orthotopically into humanized NSG mice. Five weeks after tumors were palpable, spleens and tumors were processed to a single cell suspension and were analyzed by flow cytometry. (**A**) TR-1 subset was found in the CD4 T cell subpopulation (CD45+, CD3+, CD4+) derived from spleen and tumor and (**B**) was analyzed with t-SNE maps regarding their LAG-3, CD49b and CD45Ra expression, color-coded by the expression as indicated. One representative MDA-MB-231 HTM is shown.

Figure S3.

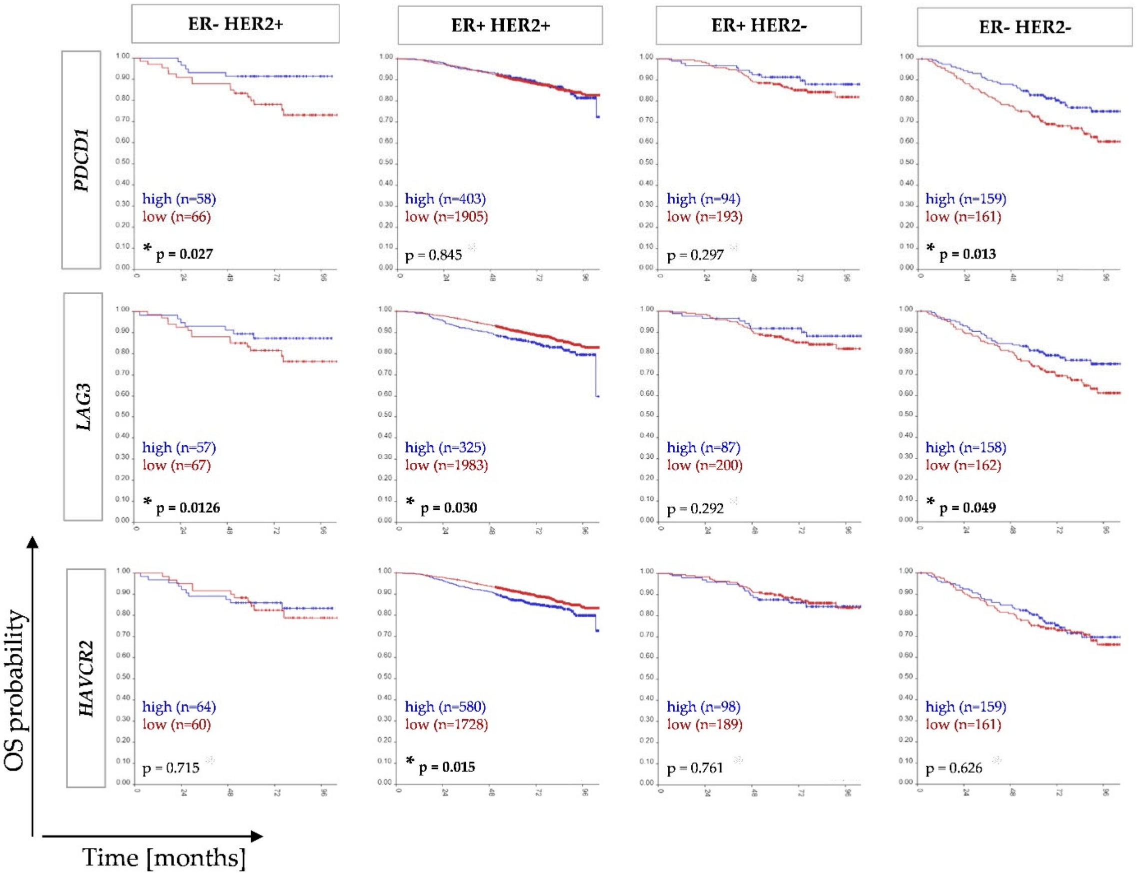

Figure S3. Overall survival of breast cancer patients according to gene expression of PD-1, LAG-3 and TIM-3. Survival curves were plotted as a Kaplan-Meier estimation curve, generated with the R2: Genomics Analysis and Visualization Platform by using the “R2: Tumor Breast (primary) - Gruvberger-Saal - 3207 - tpm - gse202203” dataset. Patients were divided into a high (red) or low (blue) expressing group, defined by the median expression of PD-1 (*PDCD1*), TIM-3 (*HAVCR2*) or LAG-3 (*LAG3*) in the TNBC patient group. OS – overall survival.
